# Novel fascial mapping of muscle spindles distribution: insights from a murine model study

**DOI:** 10.3389/fphys.2025.1571500

**Published:** 2025-05-01

**Authors:** Yunfeng Sun, Lucia Petrelli, Caterina Fede, Carlo Biz, Damiana Incendi, Andrea Porzionato, Carmelo Pirri, Xiaoxiao Zhao, Carla Stecco

**Affiliations:** ^1^ Padova Neuroscience Center, University of Padova, Padova, Italy; ^2^ Department of Neuroscience, Institute of Human Anatomy, University of Padova, Padova, Italy; ^3^ Orthopaedics and Orthopaedic Oncology, Department of Surgery, Oncology and Gastroenterology DiSCOG, University of Padova, Padova, Italy

**Keywords:** muscle spindle, fascia, perimysium, distribution, proprioception, motor control

## Abstract

Muscle spindles (MSs) are essential for proprioception and motor control. The precise distribution and localization of MSs have been the focus of major research efforts to provide a foundation for understanding their roles in various diseases and motor dysfunctions. However, there are currently disagreements on the distribution patterns of MSs, and these discrepancies hinder the advancement of novel physical therapy techniques based on MS functionality. In this study, we present an innovative fascia-based distribution pattern for MSs. Using the rat quadriceps femoris muscle as the target, serial sections of the muscle were meticulously prepared following tissue sampling, fixation, and embedding. Furthermore, four additional rat gastrocnemius and eight human muscles were processed and cut into non-successive sections by the above method. The MSs were identified and characterized using Sirius Red staining, and their locations, quantities, associated structures, and basic parameters were documented via microscopy. Our findings demonstrate that the MSs are primarily located within the fascial layers and predominantly within the perimysium; the MS capsule is structurally continuous with the perimysium and forms multiple connections in different orientations. This study demonstrates that MSs are influenced by not only changes in muscle length but also alterations in the fascia tension or state, which may have more significant impacts. Furthermore, both nerves and vessels were observed near or within the capsule of the MS but were not always presented. In some sections, no microscopically distinguishable vessels or nerve fibers were observed around the MSs. This study proposes a novel fascia-based distribution model for MSs by highlighting that MSs are embedded within the fascial matrix and that the fascia may serve as a key structural marker for locating MSs. Additionally, the structural continuity of the fascia with the MS capsule suggests its role as a potential mediator in MS functions. The present study challenges the traditional concepts of MS distribution by introducing a more refined and efficient approach for studying MSs through the fascial perspective, thereby representing a significant advancement.

## 1 Introduction

The intricate physiology of skeletal muscles involves myriad sensory structures, among which the muscle spindles (MSs) are pivotal elements (Minton, 2024). MSs are sensory receptors crucial for motor functions and proprioception; they are spindle shaped and located in almost all human muscles, with varying distributions in different muscles or species (Bhojwani et al., 2017; Bartels and Harrison, 2024). MSs sense the changes in the lengths and speeds of muscle fibers throughout the body to generate action potentials that inform the central nervous system to achieve proprioception (Banks and Proske, 2024). It is well established that MSs are implicated in many diseases as important pathological markers or potential therapeutic targets, such as guiding the injection points for botulinum toxin in spasticity (Bartels and Harrison, 2024; Kröger and Watkins, 2021; Wang et al., 2024). Numerous studies have highlighted that understanding the distribution patterns of MSs is a critical step in MS research and the development of novel interventions for proprioception (Kröger and Watkins, 2021; Wang et al., 2024; Lian et al., 2022). However, extant research conclusions on this issue are inconsistent, and none of the current conclusions can fully and thoroughly describe the distribution of MSs; this has seriously affected the development of clinical applications and scientific research of MSs.

Approximately 50,000 MSs are estimated to exist in human muscles (Banks et al., 2004), and these large numbers complicate the task of mapping MS distribution. In addition to the technological difficulties and the number of MSs, the methods for mapping MS distribution are variable with inconsistent conclusions. Previous studies have demonstrated various mapping terms, including muscle shape, muscle fiber type, nerve/vessel, and region of muscle, to address MS distribution (Bartels and Harrison, 2024; Lian et al., 2022; Papaioannou and Dimitriou, 2020; Kulkarni et al., 2001; May et al., 2022). Our previous review study (Sun et al., 2024) emphasized that MS distribution is closely associated with specific anatomical structures and muscle regions even with differences among different muscles; the typical anatomical structures comprising the most MSs are nerve ending points (NEPs), vessels, and intramuscular connective tissues (IMCT). A common pattern with respect to muscle portions (with the largest numbers of MSs) is deep > superficial/middle and proximal > distal. After comprehensively reviewing data on MS density, quantity, and distribution, there still exist some ambiguities; foremost among these is the consideration on whether there is a more stable and easier-to-observe anatomical structure that can be used to locate the MS distribution map.

The latest consensus redefines the original general fascia as the fascia system, which is a layered body-wide multiscale network of connective tissue that allows tensional loading and shearing mobility along its interfaces (Adstrum et al., 2017). The fascia system contains superficial fascia, deep fascia, visceral fascia, and neural fascia; among these, the deep fascia includes the epimysium, perimysium, and endomysium and is an important structure surrounding muscles (Adstrum et al., 2017). In the present study, the term “fascia” generally refers to the IMCT unless specified otherwise. The fascia is rich in nerve fibers, blood supply, and sensory receptors (Gatt et al., 2025). The fascia may be a reliable target to describe the distribution of MSs as it is distributed throughout the body, and the composition of fascia is closely related to the external capsule of the MS (Bordoni et al., 2025a). Studies have shown that fascia is widely used in clinical diagnosis and treatment because of its plasticity and maneuverability (Bordoni et al., 2025a; Pirri et al., 2024), but there are no available studies on the distribution of fascia and MSs till date. In the present study, the MSs from rat quadriceps femoris muscle were investigated as a series of successive slices, while four other rat gastrocnemius and eight human muscle samples were investigated via non-successive slices to highlight the topographical localization of MSs and their relationships with the fascia.

## 2 Materials and methods

The entire quadriceps femoris muscle of a rat was cut into four segments after euthanasia via sodium pentobarbital (100–200 mg/kg, intraperitoneal injection). The segments were fixed with 10% formalin solution for 24 h; following fixation in 10% neutral buffered formalin, the muscle tissues were processed for paraffin embedding according to a standard histological protocol to preserve the morphological integrity and ensure optimal sectioning quality. The fixed tissues were dehydrated using a graded ethanol series to progressively remove the water content. The samples were then transferred sequentially through 70% ethanol, 80% ethanol, 95% ethanol, absolute ethanol, and xylene. Following complete dehydration, the samples were subjected to clearing to remove ethanol and prepare the tissues for paraffin infiltration; the clearing was performed using xylene in two stages to make the tissues more transparent and compatible with paraffin. To prevent overhardening, the exposure to xylene was monitored carefully. Alternative clearing agents were also considered for environmentally sensitive protocols, but xylene was preferred owing to its superior compatibility with paraffin. The cleared tissues were immersed in molten paraffin wax (melting point: 56°C–60°C) under vacuum to replace the xylene with paraffin. The infiltration was performed twice, during which the temperature was maintained to avoid tissue shrinkage or distortion. The vacuum-assisted embedding process ensured thorough paraffin penetration, particularly in the thicker and denser tissues. After infiltration, the tissues were transferred to prewarmed embedding molds filled with molten paraffin. Then, the samples were carefully oriented to ensure the desired plane of sectioning, with the muscle fibers typically aligned perpendicular to the cutting surface. The molds were allowed to cool on a chilled metal plate to solidify the paraffin and secure the tissues.

A series of 5-µm-thick serial sections were prepared using a Leica RM2255 fully automatic rotary sectioning machine, and each section was carefully transferred onto a glass slide to enhance tissue adherence during subsequent processing. The sectioning was performed until the entire tissue within the paraffin block was exhausted, ensuring that no part of the sample was overlooked. During the process, the blade was replaced as needed to maintain section quality and avoid tissue artifacts. The sections were routinely inspected under a stereomicroscope to verify tissue integrity and alignment while minimizing fold formation and ensuring uniformity of the sections. The slides were dried overnight in an oven at 37°C to ensure proper adhesion of the sections before further histological staining and analyses. Three common staining methods were tested to decide the optimum method of identifying the MSs. By comparing Sirius Red, H&E, and Van Gieson, it was noted that the colors of different structures were easier to discriminate with Sirius Red, as shown in Figures 6D–F. Sirius Red staining was thus selected in the present study to discriminate the fascia and muscle fibers more easily; Sirius Red 0.1% staining in picric acid for 15 min showed that IMCT was presented in red while muscle fibers were presented in yellow. The MSs contain several intrafusal fibers that are thinner than the extrafusal fibers, and the intrafusal fibers are surround by a consecutive capsule, which is the basic character of MSs under the microscope. To ensure that none of the MSs were missed, the entire area of each section was analyzed in a line-by-line scan, where two authors were assigned to search for the MSs and a third author conducted microscopic investigation when an image could not be confirmed. Leica DMR and K3C microscopes were used to screen and collect the images of the MSs. ImageJ software was used to analyze the areas and lengths of the MSs. In addition to the above sample, four extra rat gastrocnemius muscles were prepared by the above steps, and non-successive sections were collected from these muscles.

Eight human muscle samples were also obtained from eight different cadavers at the Institute of Human Anatomy, University of Padova, including one sample each of the vastus lateralis, tibialis anterior, rectus femoris, fibularis brevis, and quadratus femoris muscles, along with three samples of the deltoid muscle. Six sections were obtained for each muscle sample after dehydration and fixation through the procedure described above, and the staining and microscopic investigations were consistent with those conducted for the slices of rat quadriceps femoris muscle.

The above experiments were conducted at the University of Padova; all experimentalists had relevant qualifications, and the operating procedures strictly adhered to the ethical requirements for the treatment of laboratory animals. The data visualizations and statistical analyses were conducted using PRISM 8.0.2. Student’s t-test was used to compare two groups and assess statistical significance, with the significance level set at *p* < 0.05. All related data, including the raw data and statistical results, are provided as a supplementary file and can be accessed upon request to the authors.

## 3 Results

With regard to the series of successive sections, a total of 193 sections were collected (20 from the distal, 54 from the central, and 119 from the proximal regions). These sections were observed under the microscope continuously in order, and each observed MS was recorded. Starting from the first section, each section was sequentially observed until the last section and numbered according to the order of MS appearance, positional relationships with the nerve fibers and vessels, the fascial layer in which the MS was located, and the region of the muscle, among others. The observations were made in the order of proximal, central, and distal regions of the muscle. A total of 24 MSs were successfully identified, and this result was consistent with the findings of a previous study showing a similar number of MSs in the rat hindleg muscles (Sun et al., 2024). An additional four MSs were identified from the four extra rat gastrocnemius muscles, as shown in Figure 3H.

### 3.1 General characteristics of MSs in rat quadriceps femoris muscle

**FIGURE 1:**
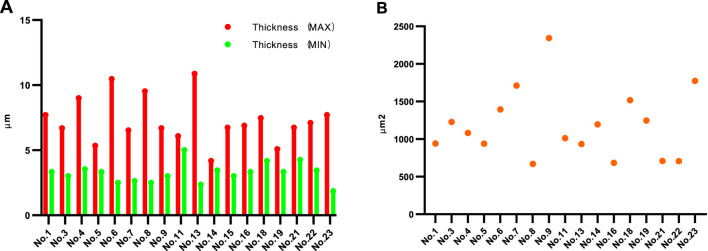
Thickness and surface area values of MSs. **(A)** Maximum and minimum thicknesses of each of the MSs. **(B)** Surface areas of the MSs. Sections within the equatorial region of the MS with intact structure and no splitting were selected to measure the thickness and area.

Eighteen MSs were eligible for calculating the capsule thickness among the 24 MSs because the capsules of six MSs were destroyed or chapped; in this measurement, the thickest and thinnest sites of each capsule were measured. The maximum capsule thickness ranged from 10.879 μm to 4.184 μm with an average of 7.277 μm, while the minimum thickness ranged from 5.021 μm to 1.871 μm with an average of 3.237 μm. The capsule thickness varied significantly for a given MS, with the differences between the maximum and minimum thicknesses ranging from 8.512 μm to 0.734 μm, as shown in Figure 1A. To calculate the surface areas, 17 structurally intact MSs were selected, and their areas varied from 2,343.33 μm^2^ to 671.09 μm^2^, as shown in Figure 1B.

### 3.2 Distribution of MSs in rat quadriceps femoris muscle

**FIGURE 2:**
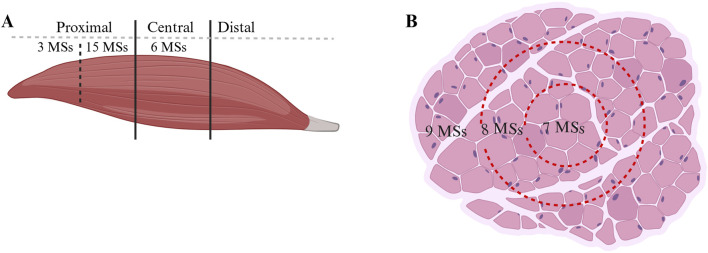
Distribution of MSs in the muscles. **(A)** Quantity of MSs in different regions in the longitudinal section. **(B)** MS quantity in the transverse section.

Differences in the MS distributions were observed across different muscle regions. In the present study, a total of 24 MSs were found in the rat quadriceps femoris muscle, of which 18 MSs were located in the proximal region and 6 MSs were located in the central region; no MSs were found in the distal region, as shown in Figure 2A. Regarding the transverse sections, each cross-section was divided into three equal areas: outer, middle, and inner areas. The distribution of the 24 MSs in the above areas was as follows: 9 MSs were located in the outer area, while 8 and 7 MSs were located in the middle and inner areas, respectively, as shown in Figure 2B.

### 3.3 Variations in the anatomical relationships between vessels/nerves and MSs

**FIGURE 3:**
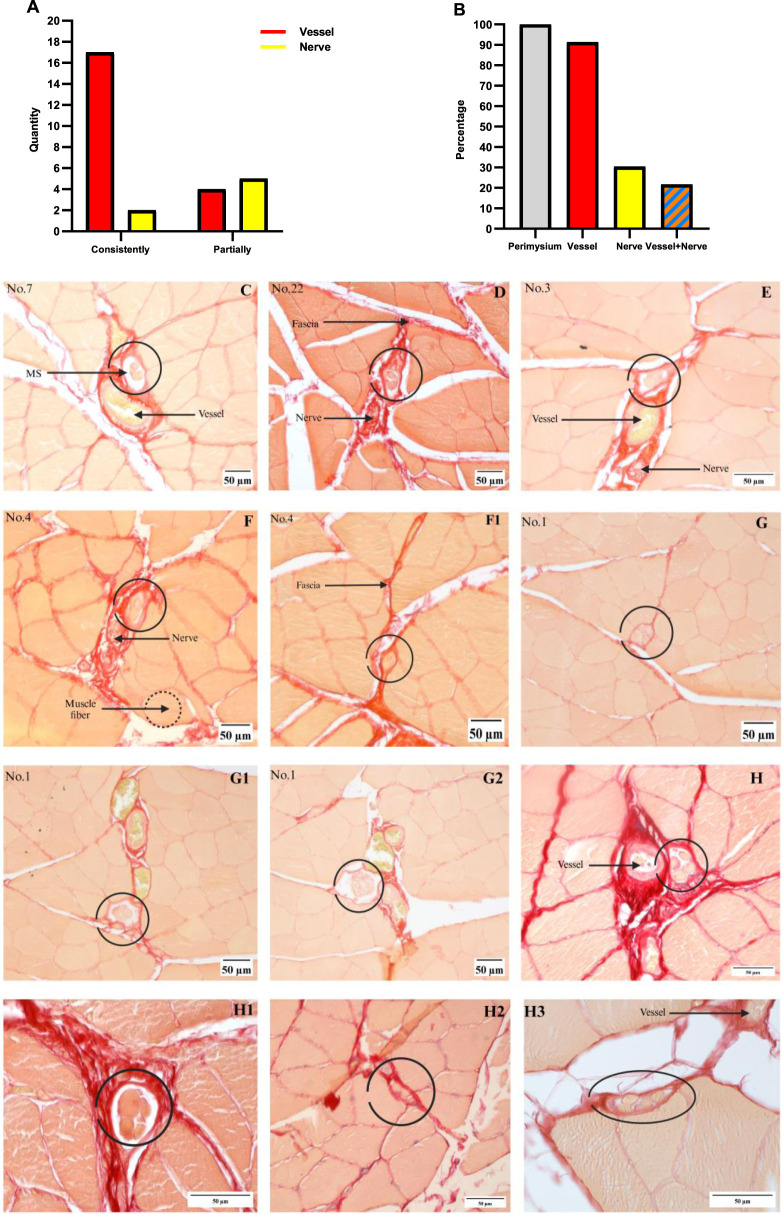
Relationships between the MSs and vessels/nerves. **(A)** Number of MSs associated with nerves or vessels; vessels: 17 MSs consistently, 4 MSs partially; nerves: 2 MSs consistently, 5 MSs partially. **(B)** The percentage of MSs present in each structure was calculated by dividing the number of MSs in the structure by 23. **(C)** MSs with only vessels. **(D)** MSs with only nerves. **(E)** MSs with both nerves and vessels. **(F, F1)** Nerves are not associated with the MS in all segments, and these pictures are from a single MS. **(G–G2)** Vessels are not associated with MSs in all segments, and these pictures are from a single MS. **(H–H3)** MSs identified from the four rat gastrocnemius muscles, where vessels are observed in **(H, H3)** and no nerve or vessel are observed in **(H1, H2)**.

Numerous studies have demonstrated that MSs are mainly bound to the nerve fibers or vessels, such that these two are considered as the landmarks of MSs. The anatomical relationships between the MSs and these two structures were recorded and analyzed for each section, as presented in Figure 3. Some MSs were accompanied by nerves or vessels or both. We calculated the numbers of MSs that were accompanied by these two structures at the current magnification, and one of the MS structured was not eligible for analysis as it was damaged. A total of 21 MSs were accompanied by vessels (17 MSs were accompanied by vessels consistently in all sections and 4 MSs were accompanied by vessels in partial sections), and seven MSs were accompanied by nerve fibers (2 MSs consistently in all sections and 5 MSs in partial sections), as shown in Figure 3A. Thus, 2 MSs were accompanied by only nerve fibers, 16 MSs were accompanied by only vessels, while 5 MSs were accompanied by both nerves and vessels. Thus, 91.3% of the 23 MSs were associated with vessels, 30.4% with nerves, and 21.7% with both structures, as shown in Figure 3B. Typical images of the MS sections are shown in Figures 3C–G. The four additional rat gastrocnemius muscles were analyzed, and three non-successive slices were collected from each sample; here, only 4 MSs were identified, of which 2 MSs were accompanied by vessels, and the other 2 MSs were not accompanied by vessels/nerves, as shown in Figure 3H.

### 3.4 Relationship between MSs and fascia

**FIGURE 4:**
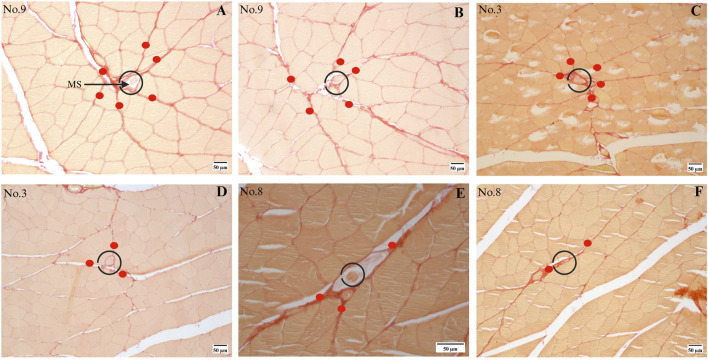
Series sections of a single MS. **(A, B)** Different slices from MS no. 9; this MS receives tension from **(A)** six or **(B)** five directions in different segments, where the directions are marked with red dots. **(C, D)** Different slices from MS no. 3. **(E, F)** Different slices from MS no. 8.

**FIGURE 5:**
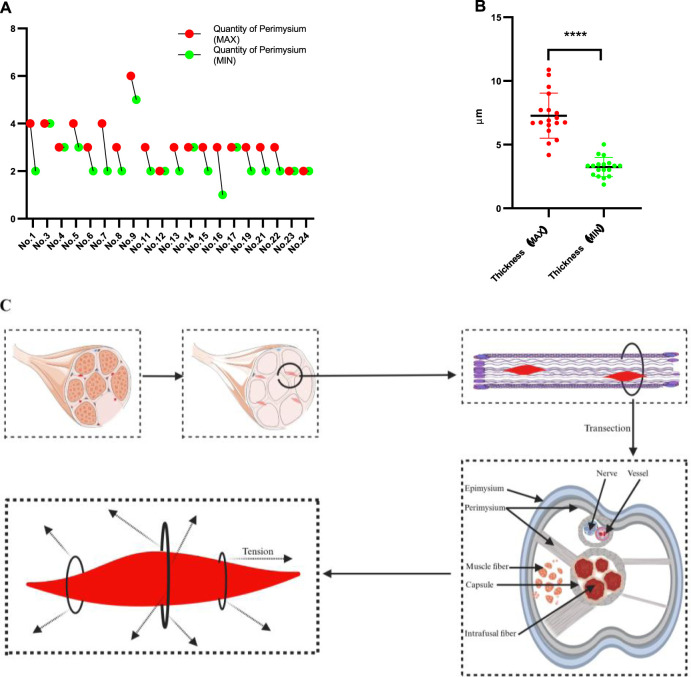
Results of the MSs identified in the rat quadriceps femoris muscle. **(A)** Number of fascia (perimysium) connections with the MS capsule; there are differences in this number between MSs and also different segments of each MS. **(B)** The comparison of thickness (maximal part of the capsule versus minimal) shows no statistical significance. **(C)** Schematic illustration of the anatomical relationship between the MS and surrounding fascia as well as the tension that it is subjected to.

Twenty out of the 24 MSs (from the quadriceps femoris) were eligible for analyzing the fascia (perimysium) connected to the capsule, while the remaining 4 MSs were ineligible owing to the destruction of the connective tissue during staining. These MSs are located in the perimysium consistently even though they are distributed in different muscle regions, as shown partially in Figure 3, and the sections of human muscle MSs supported these results as well, as shown in Section 3.6. All rat MSs were located at the intersection points of the perimysium; the number of perimysium points connected to each MS and their directions of travel were significantly different, as depicted in Figures 4, 5. More importantly, based on the observations of serial sections of the MSs, the number of perimysium points connected to each MS capsule varied at different positions of the MS, as shown in Figures 4A–F and detailed in Section 3.5.

### 3.5 MSs are affected by the perimysium for different dimensions

The above results demonstrate that the MSs are primarily distributed in the perimysium, and further investigations reveal that the MSs are located at the intersection points of the perimysium arriving from different directions; however, there is diversity among the different regions even in the same MS. The maximum and minimum numbers of the perimysium points connected to each MS and arriving from different directions are shown in Figure 5A, where the maximum number ranges from 2 to 6 while the minimum ranges from 1 to 5. To understand whether the number of perimysium points connected to the capsule is correlated with the thickness of the capsule, a test was conducted, which showed there was no association between these parameters. The comparison between maximum and minimum thickness showed a significant result, as presented in Figure 5B, suggesting that the thickness of the MS capsule is variable. However, the sample size of the present study is limited, and our results are not sufficient to confirm the above relationship. Even so, these results suggest that the MSs may receive multidirectional tension from the fascia and that the directions and amount of tension are unpredictable. The statuses of the MSs in the fascia are summarized and presented in Figure 5C.

### 3.6 MSs are located in the fascia in human muscles

**FIGURE 6:**
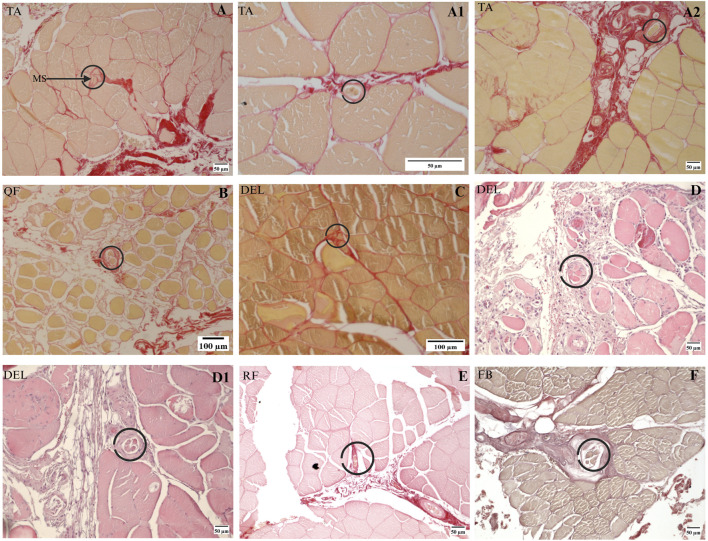
MSs in human muscles. **(A–A2)** Three MSs were found in one human TA muscle sample. **(B)** A single MS was found in the human QF muscle sample. **(C)** A single MS was found in one of the deltoid muscle samples. **(D, D1)** Two MSs in another deltoid muscle sample based on H&E staining. **(E, F)** MSs found in one rectus femoris and one fibularis brevis, where **(E)** is based on Sirius Red staining and **(F)** is based on Van Gieson staining. DEL, deltoid; FB, fibularis brevis; QF, quadratus femoris; RF, rectus femoris; TA, tibialis anterior.

In the present study, MSs from human muscles were investigated by collecting non-successive sections using the procedures described in Section 2. Ten MSs were identified successfully from seven muscle samples; all MSs were located in the perimysium, consistent with the results from the rat muscle, except for one MS in the tibialis anterior muscle that was located in the endomysium, as shown in Figure 6A. The capsule of the MSs is connected to the perimysium in different directions as well. As illustrated in Figure 6, the MSs may be associated with vascular or neural structures or even be independent of such associations. However, the number of human muscle samples and MSs identified in this study are limited given the large sizes of human muscles that prevented continuous sectioning. Therefore, the relationship between human MSs and fascia remains incompletely understood in this study.

## 4 Discussion

MSs are widely distributed in the body and have important physiological functions (Wang et al., 2024; Yan et al., 2025) that warrant further clarification of their distribution patterns to enhance clinical applications. However, numerous studies have previously demonstrated that the distribution of MSs in the muscles is variable (Sun et al., 2024). The terms “muscle region, nerve fiber, vessels, and inner/middle/outer layer” have all been used to describe the distribution of MSs in previous studies according to our earlier review (Sun et al., 2024); however, none of these terms can be considered as unique or containing the entire MS without being combined with others, so the disunity and variety of MS distribution patterns have led to restrictions on MS-related applications in the clinic. The findings of the present study suggest that some MSs have no visibly associated vessels or nerves under the microscope or that these structures are only present in partial sections of the MS; this finding is a good explanation for why the distribution of MSs based on nerves/vessels has always yielded inconsistent conclusions.

In the present study, a fascia-based localization and distribution pattern is reported and investigated for MSs. To the best of the authors’ knowledge, this work is a pilot effort to provide a detailed and accurate description of the localization of MSs; the fascia-based pattern considered herein not only offers a more concrete framework for MS distribution but also opens avenues for fascia-based treatments in MS-related diseases. The present study first demonstrates that all MSs found in rat muscles are located in the perimysium exclusively and that the MSs are only embedded in the fascia (perimysium) and merge with the fascia through the capsule. In addition to the complete interpretation of this feature through experiments in this study, existing anatomical studies are also known to support these findings. Intrafusal fibers are enfolded by the capsule and oriented parallel to the extrafusal fibers; the classical anatomical features of MSs indicate that the MS must be connected to the IMCT, but previous studies have not clarified the fascia layer involved. Thus, our findings are aligned with existing anatomical knowledge of MSs. There were large gaps in fascia and MS research for a long period of time, and only a few studies have mentioned the relationships between the MSs and fascia system. The study by James et al. (2022) in sheep multifidus muscle demonstrated that 59.2% of the MSs were close to a major fascial element, while another earlier study by Kubota and Masegi (1972) in the Japanese shrew-mole claimed that the MSs were strongly close to and along the distinct collagenous septum. These studies suggest that the fascia-based pattern exists in not only rats but also other species. Similar studies in different species are therefore necessary in the future.

Beyond these observations, another remarkable finding in the present study is that the capsule of the MS is attached to the fascia (perimysium) in different directions; the proportion of the perimysium connected to the capsule varies for different MSs and even for different segments of a single MS. Since the MSs are attached to the perimysium in several directions, our interesting results demonstrate that the tension of the fascia may affect the status of the MSs from various directions or even in different segments of a single MS, as presented in Figure 5C. The above anatomical relationship indicates that the MSs experience a complex biomechanical environment mainly involving the fascia but not muscle fibers. Studies by Bordoni et al. (2025b) and Kassolik et al. (2013) prove that local lesions lead to global modifications of the fascia tension, implying that the MSs may be affected by any local lesion in the global fascia system, which in turn results in abnormal proprioception; the present study offers a potential mechanism for this issue. The results from rat muscles demonstrate that MSs are mainly embedded in the fascia (perimysium) and located at the intersection points of the perimysium. The histological image analysis of a small number of human MSs also tended to support a similar conclusion. Ten MSs were found from seven human muscles, of which nine were embedded in the perimysium; only one MS was found to be connected to the endomysium, and the capsule-connected endomysium was thicker, as shown in Figure 6A. Therefore, in the present study, it was challenging to identify the specific fascia layer from which MSs manifest in human muscles.

Fascia refers to the entirety of connective tissues surrounding the body that has excellent biomechanical properties and is operable using special technology (Barič et al., 2024). The above results prove that MSs may be affected by the fascia in various directions and that the function and sensitivity of a MS may be modified even with very little tension in any single direction. Normalizing the tension of the entire fascia in different directions is a feasible and promising method to maintain healthy MSs. This hypothesis corresponds to the mechanism of fascia manipulation, which has been applied in clinics with notable effects (Isaji et al., 2024). Fascia manipulation normalizes the tension of the fascia in all layers such that the effects are felt in both local tissues and the entire body (Isaji et al., 2024; Tozzi, 2012). In addition, fascia manipulation also benefits blood support and the tension of nerve fibers (Roman et al., 2024; Schleip, 2003), so further studies on the effects of fascia manipulation on the MSs will be an important research area to promote future clinical applications of MSs. Previous studies showed that impaired dynamic balance and increased peroneal reaction time were common in chronic ankle instability, following which the fascia thickness increased (Brighenti et al., 2023; Pirri et al., 2021); hence, it is suggested that MSs may play vital roles during ankle injury and consequent dysfunctions based on the fascial MS distribution pattern. Other studies on lower back pain and knee ligament injuries suggest that the fascia and proprioception are involved in these lesions (Benítez-Martínez et al., 2023; Prados-Barbero et al., 2024); our findings show that the fascia and fascia-based MS distribution may thus have potential clinical value.

The present study has some notable limitations. First, we did not specifically investigate the relationship between muscle fiber types and MS distribution. Therefore, this study does not address whether the fascia-based MS distribution model is superior to a muscle-fiber-type-based MS distribution model; future research should aim to explore this aspect more comprehensively using fiber-type staining or electrophysiological assessments. Second, the sample size used in this study was small; although the findings in rats support the conclusions, additional studies are needed to strengthen the evidence. Third, the conclusion lacked strong evidence with regard to human muscles, so further studies focusing on various muscle types are urgently needed.

## 5 Conclusion

The present study demonstrates that MSs embedded in the perimysium of the fascia are dominant and that the perimysium plays an important role in the distribution of the MSs, as MSs are located at the intersection points of the perimysium. There are significant differences in the area of the perimysium connected to the MS capsule, and these differences exist for different MSs and also for different segments of a single MS. The techniques and theory of MS localization based on the fascia (perimysium) presented herein are more efficient and accurate than existing methods. In addition, this study suggests that improving the MS functions by regulating the fascia to improve proprioception is an important research direction for the future. The findings of this work are of great significance to promote the development of the fascia and clinical applications of MSs.

## Data Availability

The original contributions presented in the study are included in the article/Supplementary Material; further inquiries can be directed to the corresponding author.
